# Antibiotic-impregnated articulating cement spacer maintained for 7 years in situ for two-stage primary total knee arthroplasty: a case report

**DOI:** 10.1186/s12891-019-2571-7

**Published:** 2019-04-25

**Authors:** Yong-Beom Park, Chul-Won Ha, Jae Won Jang, Manyoung Kim

**Affiliations:** 1 Department of Orthopedic Surgery, Chung-Ang University Hospital, Chung-Ang University College of Medicine, 102 Heukseok-ro, Dongjak-gu, Seoul, 06973 South Korea; 2 Department of Orthopaedic Surgery, Samsung Medical Center, Sungkyunkwan University School of Medicine, 81 Irwon-ro, Gangnam-gu, Seoul, 06351 South Korea; 3 Stem Cell and Regenerative Medicine Institute, Samsung Medical Center, 81 Irwon-ro, Gangnam-gu, Seoul, 06351 South Korea; 4 Department of Health Sciences and Technology, SAIHST, Sungkyunkwan University, 81 Irwon-ro, Gangnam-gu, Seoul, 06351 South Korea; 5 Department of Orthopedic Surgery, Yonsei Knee and Spine Hospital, 568 Cheonho-daero, Gwangjin-gu, Seoul, South Korea; 6 Department of Orthopedic Surgery, The Leon Wiltse Memorial Hospital, 560, Gyeongsu-daero, Dongan-gu, Anyang-si, Gyeonggi-do 14112 South Korea

**Keywords:** Total knee arthroplasty, Infection, Articulating spacer, Retention

## Abstract

**Background:**

Antibiotic-impregnated articulating cement spacers can maintain interim joint motion with the potential to enhance functional status and improve patient satisfaction. Articular surfaces with cement against cement have raised concerns regarding mechanical complications and cement debris during knee motion. However, long-term clinical conditions regarding these concerns are not well addressed.

**Case presentation:**

We report a case in which articulating cement spacers were maintained in situ for 7 years. The patient had severe left knee pain with an ankylosing knee and severe tricompartmental arthritis due to tuberculous infection. We planned to perform one- or two-stage primary total knee arthroplasty (TKA), depending on the presence of infection. Persistent osteomyelitis was found intraoperatively. The second-stage TKA was delayed on the patient’s request. As the patient was satisfied with the improved knee function and pain relief after using articulating cement spacers. No symptom or sign that suggested recurrent infection or systemic toxicity was found during the 7-year follow-up. However, it seemed that the bone loss progressed insidiously. At the 7-year follow-up, a broken articulating cement spacer and medial femoral condylar fracture were found. The second-stage TKA was performed, and a considerable amount of bone loss surrounded by dense granulation tissue was observed intraoperatively. Excisional biopsy of the tissue revealed chronic foreign body reaction with infiltration of giant cells and macrophages.

**Conclusion:**

Although the articular spacers were maintained for 7 years without major complications, regular observation of the development and progress of bone loss was required. Surgeons should take considerable bone loss into account during conversion TKA in patients with a prolonged retention of articulating cement spacers.

## Background

The usefulness of antibiotic-impregnated articulating cement spacers has been extensively studied for the treatment of infections after two-stage revision total knee arthroplasty (TKA) [1–13]. It is also used in two-stage primary TKA to treat advanced knee arthritis with coexistent joint infection [14]. Static spacers can lead to prolonged restriction of knee motion with limited knee function [4, 15, 16]. However, articulating spacers can be designed to allow range of motion and protect weight bearing between stages [1, 3, 11]. Compared with static spacers, articulating spacers have shown greater range of motion at final follow-up [2, 4, 5, 8–10], less bone loss between stages [2, 4, 8, 9], and decreased difficulty in exposure during reimplantation [2, 8–10, 12]. In addition, the currently available evidence suggests that articulating spacers have similar or better outcomes than static spacers in infection control [2, 5, 7–10, 17].

Articulating spacers have gained popularity owing to these advantages. However, few studies have reported retention of articulating spacers in infected TKA [5, 18, 19]. In addition, detailed clinical information on long-term follow-up of retained articulating cement spacers is limited. Articular surfaces with cement against cement cause concern with regard to mechanical complications and cement debris during knee motion in long-term clinical conditions. Here, we report a case of in situ maintenance of articulating cement spacers during the interim period of two-stage primary TKA for 7 years. We adhere to CARE (CAse REport) Statement and Checklist for accurate reporting [20].

## Case presentation

**Fig. 1:**
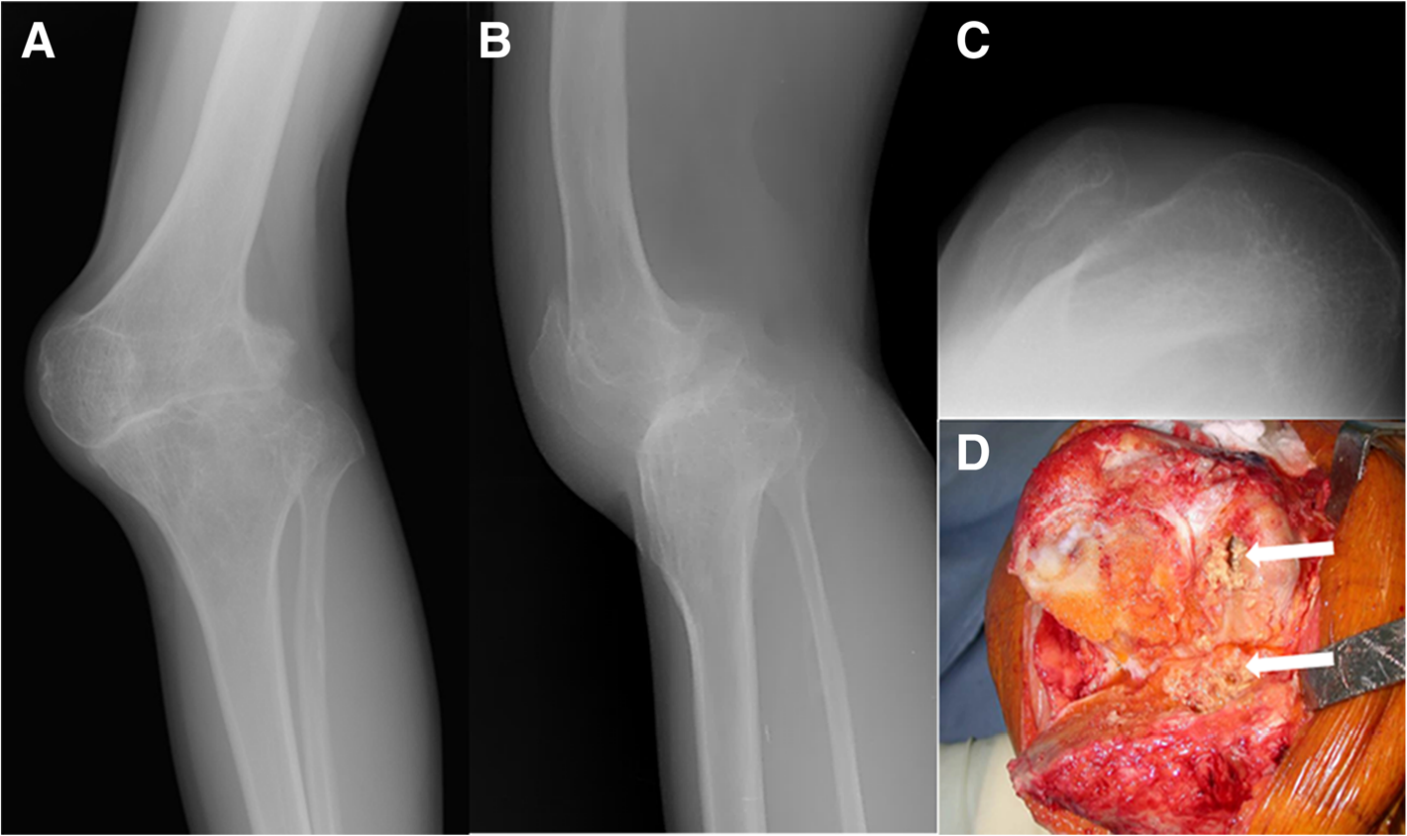
**a–d** Preoperative knee radiographs in the anteroposterior (**a**), lateral (**b**), and merchant views (**c**), showing tibiofemoral ankylosis with deformity and tricompartmental arthritis. (**d**) An intraoperative photograph taken after takedown of fusion, showing necrotic bone (arrow) in the lateral femoral condyle and lateral tibial plateau

A 44-year-old man was admitted to our clinic because of left knee pain. He had pulmonary tuberculosis at 6 years old. He presented pus-forming arthritis, which was presumably tuberculous arthritis, in the left knee after 2 years, with spontaneous remission after closure of the draining sinuses. Thereafter, no recurrent symptom of infection was observed. However, the deformity and growth disturbance progressed with the knee pain. He had limb lengthening and alignment correction for the leg length discrepancy and genu valgum. However, his left knee pain continued despite the deformity correction. Radiographs showed a fused knee with severe tricompartmental arthritis (Fig. 1). Severe limitation in range of motion was observed on the left knee. We planned to perform one- or two-stage primary TKA depending on the presence of infection [14].

**Fig. 2:**
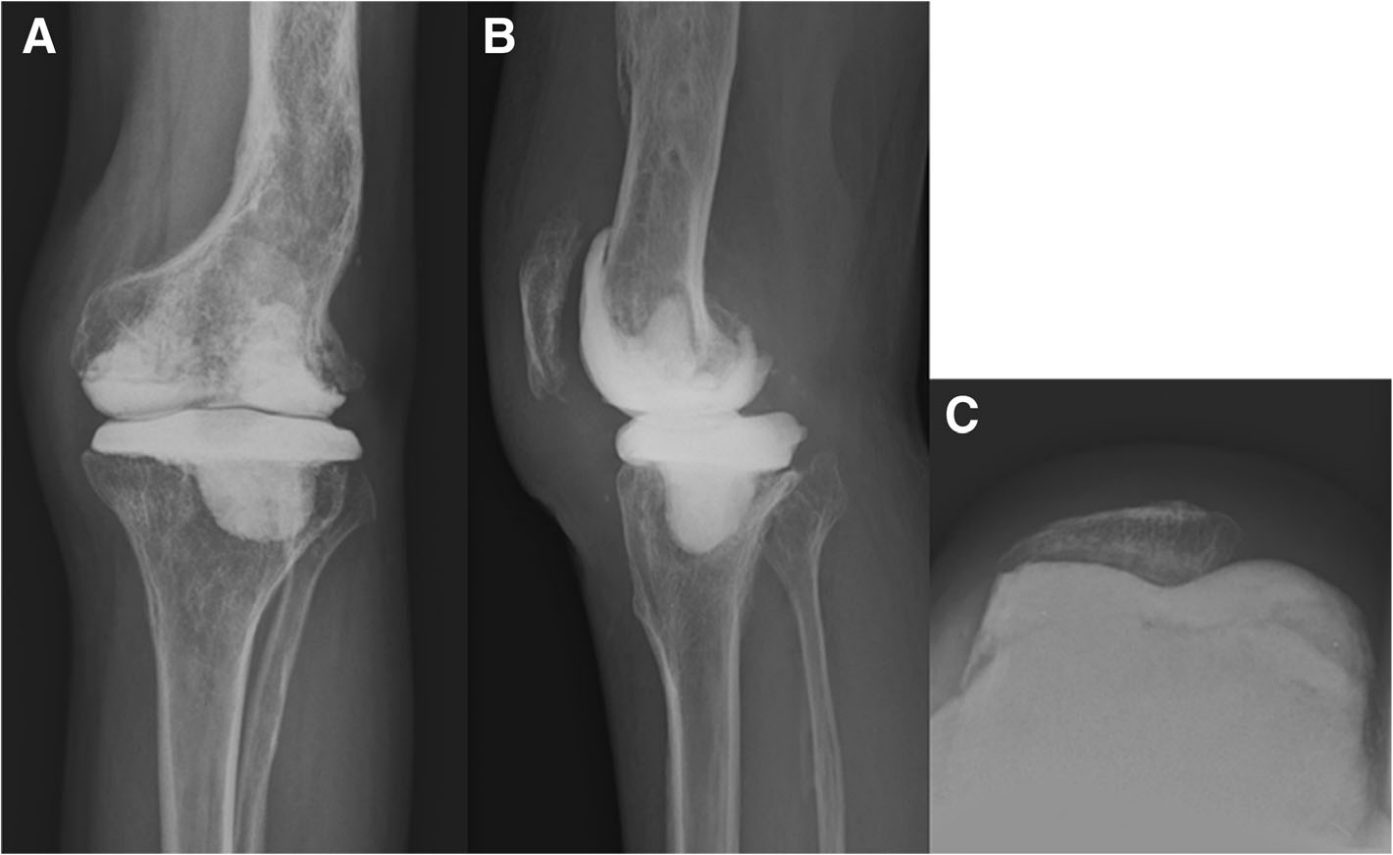
**a–c** Postoperative anteroposterior (**a**), lateral (**b**), and merchant knee radiographs (**c**) taken at 6 weeks after the implantation of articulating spacers. The medullary protruded portion of the tibial articulating spacer was deviated to the lateral side because of the contained bone defect of the lateral tibial plateau after debridement of the infected bone. The contained bone defect was also left in the lateral femoral condyle after debridement of the infected bone

Intraoperatively, a large subchondral abscess was found in the lateral femoral condyle and lateral tibial plateau after takedown of the fusion (Fig. 1d). On the basis of the necrotizing inflammation with granuloma in the frozen-section biopsy, active tuberculosis was suspected. Aggressive debridement and curettage of the infected and necrotic bone and soft tissues were performed.. After bone cuts and soft tissue balancing to prepare for TKA, articulating cement spacers (vancomycin 4 g and streptomycin 2 g per 1 batch) were made intraoperatively and applied to the tibial and femoral sides in sequence using intraoperative cement molds with a previously described technique [14, 21] (Fig. 2). Relative medial and lateral stabilities were confirmed intraoperatively after inserting the articulating cement spacers. The diagnosis of tuberculosis infection was confirmed by isolating *Mycobacterium tuberculosis* from cultures. We decided to delay the TKA for at least 6 to 9 months to allow the administration of antituberculous drugs [22]. Evaluation at 1-year follow-up revealed no recurrent infection after sufficient antituberculous drug treatment. Therefore, we recommended TKA surgery as planned. However, the patient was comfortable with the articulating cement spacers. He refused a conversion to TKA for personal reasons. At every visit thereafter, he consistently wanted to delay the second-stage surgery.

**Fig. 3:**
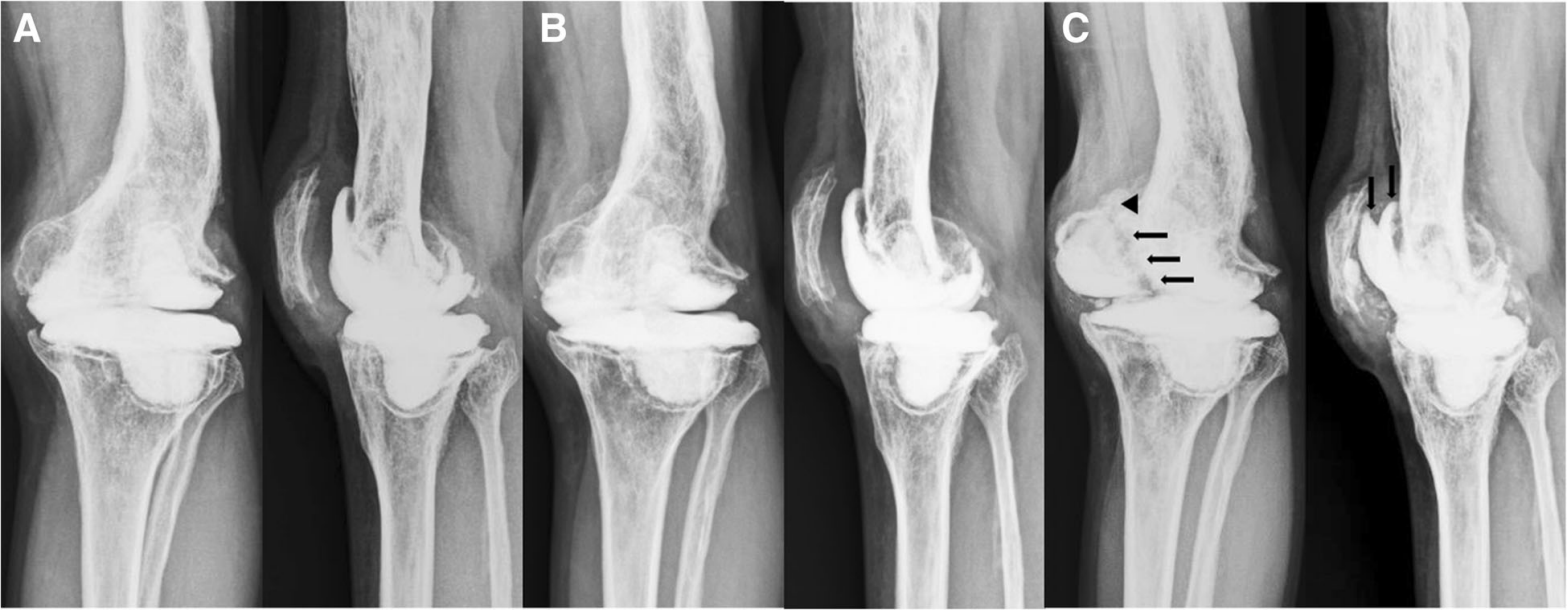
**a–c** Postoperative anteroposterior and lateral knee radiographs taken at 5 years (**a**), 6 years (**b**), and 7 years (**c**) after the implantation of articulating spacers. (**a**, **b**) Radiolucent lines along the bone-cement interface. (**b**) The longitudinal split fracture of the femoral articulating spacer component (arrow) with cortical breakage of the medial femoral condyle (arrowhead) observed at 7 years after the implantation of articulating spacers

**Fig. 4:**
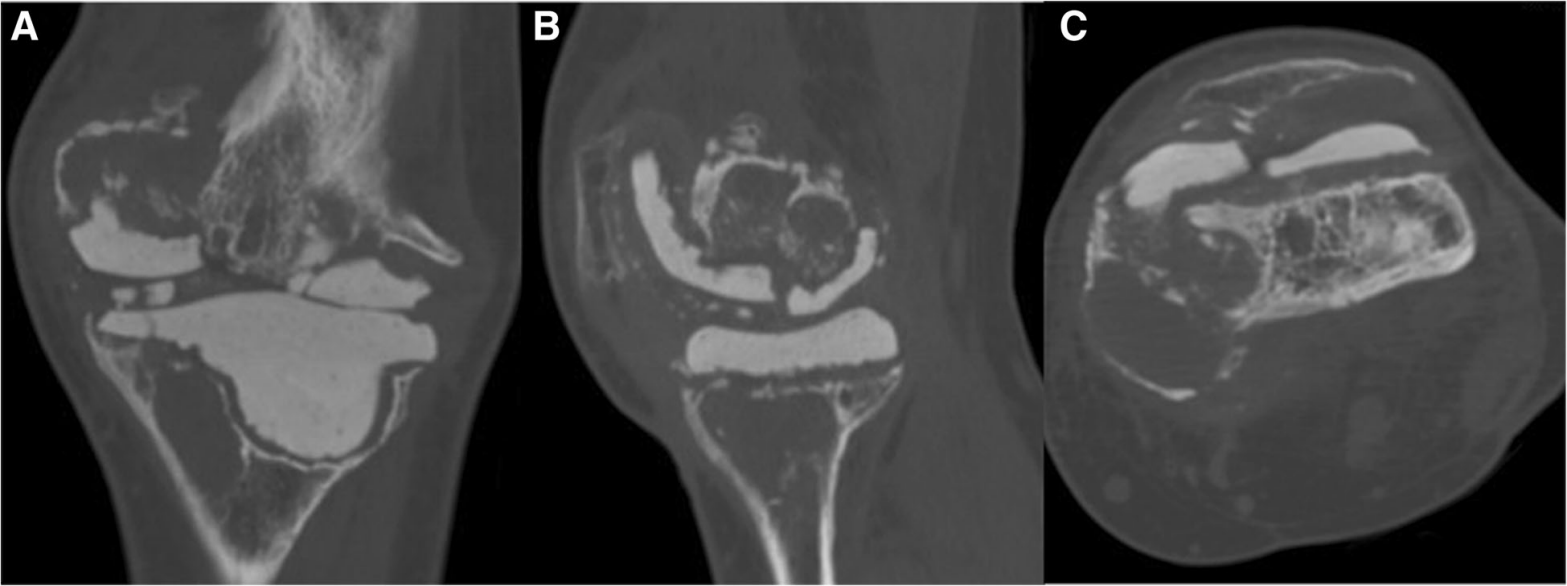
**a–c** Left knee computed tomography scans of the coronal (**a**), medial sagittal (**b**), and femoral axial planes (**c**) at 7 years after the implantation of articulating spacers, showing the underlying bone loss around the femoral and tibial components of the articulating spacers. It was more severe in the medial femoral condyle and medial tibial plateau. Cortical thinning and discontinuity of the medial femoral condyle could be observed

Painless activities were possible, including gait, step ascent and descent, and rising from a chair with full load bearing. No evidence was found that suggested recurrent infection or systemic toxicity. Laboratory markers of infection, and renal and hepatic toxicities were followed up once in a year. No abnormal finding was identified. A slowly progressing small bone loss was observed at the bone-cement interface. However, no notable mechanical problem such as subluxation, dislocation, periprosthetic, or implant fracture were observed on the serial knee radiographs (Fig. 3a and b). At the 7-year follow-up, the patient complained of left knee pain for 2 months. Radiographic and computed tomographic evaluations revealed collapse of the medial femoral condyle with fracture of the femoral articulating spacer component (Figs. 3c and 4).

**Fig. 5:**
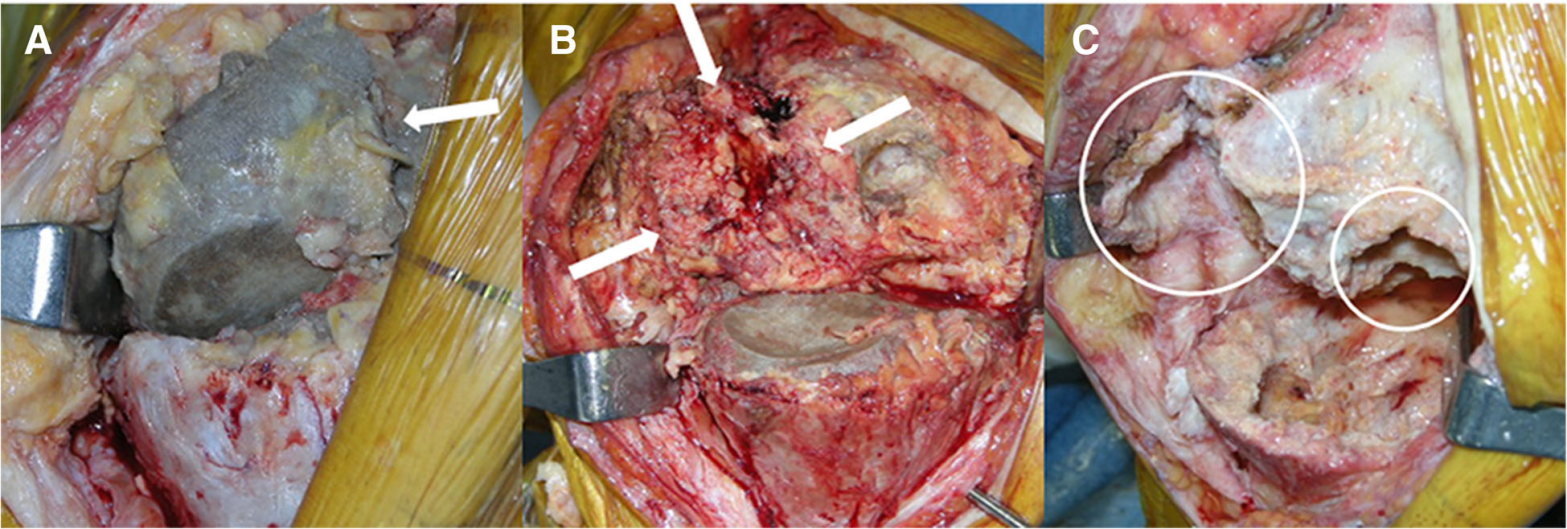
**a–c** (**a**) Longitudinal split fracture of the femoral component of the articulating spacer (arrow) found intraoperatively. (**b**) After removal of the femoral spacer component, a dense layer of fibrous tissue surrounding the femoral bony surface was found, particularly in the medial femoral condyle (white arrow). The tibial component of the articulating spacer was intact. (**c**) After meticulous debridement of the soft tissue and bone, considerable amounts of bone loss can be found in the medial femoral condyle (large circle) as compared with the lateral femoral condyle (small circle)

**Fig. 6:**
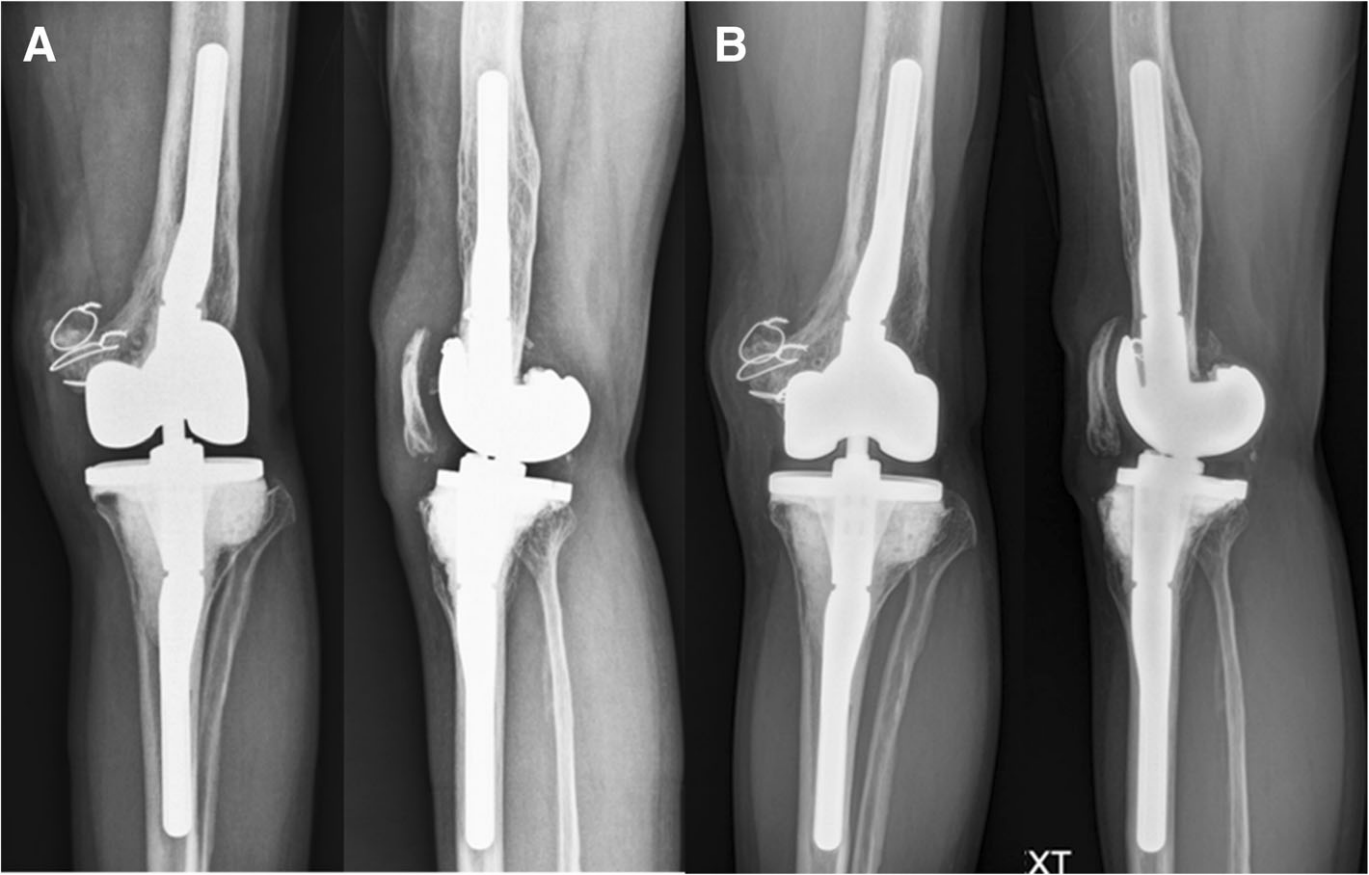
**a, b** Postoperative anteroposterior and lateral knee radiographs taken at 6 weeks (**a**) and 2 years (**b**) after the second-stage surgery

**Fig. 7:**
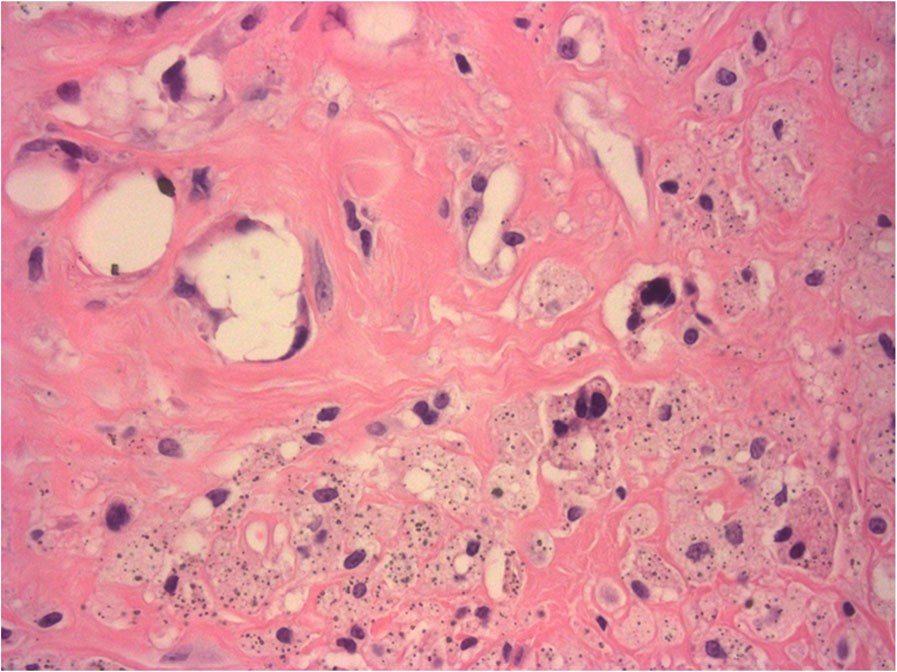
Photomicrograph of foreign body granulation tissue showing variable sizes of negative patterns of polymethyl methacrylate (PMMA) particles surrounded by giant cells and macrophages. PMMA particles are well known to dissolve during normal preparation for paraffin embedding. Tiny particles were assumed to be abraded X-ray contrast medium used as an ingredient in bone cement. They were accumulated in phagocytes with sizes ranging from 0.5 to 1 μm in diameter. Marked fibrous tissue surrounding the inflammatory cells could be observed (hematoxylin and eosin staining; original magnification, × 400)

At 7 years after the implantation of spacers, we converted the articulating spacers to TKA using the NexGen Legacy Constrained Condylar Knee system (Zimmer, Warsaw, IN, USA). The appearance of the knee joint and the finding from the intraoperative frozen-section examination suggested no residual infection. The femoral component of the cement spacer, along with the imaging study, showed a bisecting vertical fracture (Fig. 5a). The tibial component was intact (Fig. 5b). After removing the cement spacers, the dense fibrotic granulation tissue surrounding the bone surface was exposed, especially in the medial femoral condyle (Fig. 5b). Excisional biopsy of the tissues was performed. Considerable bone loss with cortical bone breakage was observed in the medial femoral condyle after the debridement of the granulation tissue and necrotic bone (Fig. 5c). We conducted wiring for the cortical bone breakage, and an allogenous morselized bone graft was performed for the bone loss. Augmentation blocks were additionally used for the remnant bone defect. In the proximal tibia, the considerably contained bone defect was restored with an allogenous morselized bone graft (Fig. 6a). Histological examination of the specimens from the bone surface of the medial femoral condyle showed marked fibrosis with a significant foreign body reaction characterized by infiltration of giant cells and macrophages (Fig. 7). Direct visualization of polymethyl methacrylate (PMMA) particles was impossible because PMMA in the bone cement was dissolved in the organic solvent used during the routine histological processing. However, a negative pattern of the surrounding foreign body giant cells was observed, with various sizes [23, 24]. Many tiny particles were assumed to be abraded zirconium dioxide, an X-ray contrast medium used as an ingredient in the Palacos R bone-cement [25]. These particles were mainly located within the cytoplasm of mononuclear macrophages. No evidence of infection or malignant lesion was found.

At the 2-year follow-up, the patient had a passive range of motion from 0° to 90°, with stability. TKA was maintained without osteolysis (Fig. 6b). He was satisfied with the clinical outcome after TKA conversion.

## Discussion and conclusions

This paper reports a case of in situ maintenance of articulating cement spacers for approximately 7 years. Only a few studies have reported retention of cement spacers for TKA-related infection [5, 18, 19]. Two studies used re-sterilized prosthesis spacers to treat the infection [5, 18]. The presence of a metallic or plastic component could favor bacterial adhesion [26]. In addition, articulating cement spacers could contain a relatively larger amount of antibiotic power than re-sterilized prosthesis spacers. Although one study has used cement spacers, detailed clinical information regarding the retention of cement spacers has not been documented [19]. Therefore, this is the first case report that investigated the detailed clinical outcome of a patient with retained articulating cement spacers. In this case, no specific functional problem, reinfection, systemic toxicity, or malignant lesion were observed during a long period between the stages. However, the underlying periprosthetic bone loss observed in the second-stage surgery, especially in the medial femoral condyle, seemed to have progressed insidiously, possibly causing the main problem of the long-term maintenance of articulating cement spacers in this case [27]. However, evidence of bone loss was not definite on the serial radiographs except at the bone-cement interface. Plain radiographs tend to underestimate the presence and extent of bone resorption [28, 29]. Several possible reasons can explain bone loss. First, cement spacers themselves could be the cause of periprosthetic bone loss due to micromotion. The amount of bone loss has been reported to be associated with the length of time after implantation of cement spacers [9]. In this case, the radiolucent lines along the bone-cement junction implied micromotion of the articulating spacers, although no remarkable evidence of migration was found in the serial knee radiographs. Second, progressive medial or lateral laxity could be a possible cause of bone loss through uneven stress distribution, which could increase the compressive load to the medial side of cement spacers and underlying cancellous bone. The load concentration on the medial compartment might be relevant to the bone loss concentrated on the medial femoral condyle and the feature of the longitudinal split fracture in the femoral component of the cement spacer. However, a considerable amount of bone loss could not be fully explained by these mechanical factors. Foreign-body reaction induced by cement debris might be a possible cause of severe bone loss. In this case, the dense layer of fibrotic connective tissue at the bone-cement interface revealed chronic foreign-body reaction, with phagocytes accumulating tiny particles assumed to be abraded zirconium dioxide. Bone resorption has been reported to be associated with foreign-body reaction induced by the radiopaque agent of bone cement [30, 31]. Abraded cement debris can be produced at cement-against-cement articulation [25, 32]. Long-term maintenance of articulating cement spacers could be problematic because of the accumulation of tiny particles. Therefore, in cases of delayed second-stage surgery, regular and precise observation about the development and progress of bone loss is needed. Follow-up with computed tomography could aid the diagnosis and measurement of bone loss to assess the risk of mechanical complications and level of difficulty of the second-stage surgery.

Several factors might have caused the long-term survival of the articulating cement spacers in this case despite that the patient was allowed to perform tolerable load-bearing tasks. First, the patient did not have coronal instability or uncontained bone defects after the first-stage surgery. Motions with articulating cement spacers are suggested to be unsuitable for patients with severe bone loss, ligamentous instability, or extensor mechanism problem [6, 33]. Second, this patient had mild weakness of the ipsilateral lower limb. Therefore, high-level physical activity could be limited. Third, our intraoperative construction technique for articulating cement spacers enables inter-digitation between the irregular bone surface and the cement spacer with an adjustable extension and flexion gap [14, 21]. We applied an intraoperatively manufactured mold to a bolus of bone cement that was put on the irregular bony surface of each femoral and tibial articular sides in the late doughy phase. After a tibial cement spacer was constructed, the thickness of the femoral cement spacer was adjusted to make ideal extension and flexion gaps with the tibial cement spacer. During recurrent knee extension and flexion, the deformed surface of the femoral cement spacer during knee motion was restored by reapplying the mold. Intraoperatively, a high degree of stability was possible in the interface between the bone and the articulating cement spacer or between the femoral and tibial components of the articulating cement spacer, although some lateral laxity unavoidably progressed slowly thereafter.

In this case, the patient had maintained the articular spacers in the left knee in situ for approximately 7 years without major functional problems, reinfection, systemic toxicity, or mechanical complications, despite allowing tolerable load-bearing tasks. The bone loss progressed insidiously, which might have caused difficulties in the second-stage surgery, with probable risk in the development of mechanical problems. Therefore, regular and precise observation about the development and progress of bone loss is required with radiographsor computed tomography in doubtable case. Care should be taken regarding a considerable amount of bone loss in conversion TKA in patients with prolonged retention of articulating cement spacers.

## Abbreviations

PMMA: Polymethyl methacrylate
TKA: Total knee arthroplasty
